# Emphasizing the Operational Role of a Novel Graphene-Based Ink into High Performance Ternary Organic Solar Cells

**DOI:** 10.3390/nano10010089

**Published:** 2020-01-02

**Authors:** Minas M. Stylianakis, Dimitrios M. Kosmidis, Katerina Anagnostou, Christos Polyzoidis, Miron Krassas, George Kenanakis, George Viskadouros, Nikolaos Kornilios, Konstantinos Petridis, Emmanuel Kymakis

**Affiliations:** 1 Department of Electrical & Computer Engineering, Hellenic Mediterranean University (HMU), Estavromenos, 71410 Heraklion, Greece; kosdimitris@hmu.gr (D.M.K.); katerinanag@hmu.gr (K.A.); polyzoidis@hmu.gr (C.P.); kmiron@hmu.gr (M.K.); viskadouros@hmu.gr (G.V.); kornil@hmu.gr (N.K.); c.petridischania@gmail.com (K.P.); 2 Department of Materials Science and Technology, University of Crete, 71003 Heraklion, Greece; 3 Institute of Electronic Structure and Laser, Foundation for Research and Technology-Hellas, N. Plastira 100, 70013 Heraklion, Greece; gkenanak@iesl.forth.gr; 4 Department of Mineral Resources Engineering, Technical University of Crete, 73100 Chania, Greece; 5 Department of Electronic Engineering, Hellenic Mediterranean University (HMU), 73132 Chania, Greece

**Keywords:** ternary organic solar cells, graphene ink, functionalization, air-processed, cascade effect, charge transfer

## Abstract

A novel solution-processed, graphene-based material was synthesized by treating graphene oxide (GO) with 2,5,7-trinitro-9-oxo-fluorenone-4-carboxylic acid (TNF-COOH) moieties, via simple synthetic routes. The yielded molecule N-[(carbamoyl-GO)ethyl]-N′-[(carbamoyl)-(2,5,7-trinitro-9-oxo-fluorene)] (GO-TNF) was thoroughly characterized and it was shown that it presents favorable highest occupied molecular orbital (HOMO) and lowest unoccupied molecular orbital (LUMO) energy levels to function as a bridge component between the polymeric donor poly({4,8-bis[(2-ethylhexyl)oxy]benzo[1,2-b:4,5-b′]dithiophene-2,6-diyl}{3-fluoro-2-[(2-ethylhexyl)carbonyl] thieno[3,4-b]thiophenediyl}) (PTB7) and the fullerene derivative acceptor [6,6]-phenyl-C_71_-butyric-acid-methylester (PC_71_BM). In this context, a GO-TNF based ink was prepared and directly incorporated within the binary photoactive layer, in different volume ratios (1%–3% ratio to the blend) for the effective realization of inverted ternary organic solar cells (OSCs) of the structure ITO/PFN/PTB7:GO-TNF:PC_71_BM/MoO_3_/Al. The addition of 2% *v*/*v* GO-TNF ink led to a champion power conversion efficiency (PCE) of 8.71% that was enhanced by ~13% as compared to the reference cell.

## 1. Introduction

Due to the highly increased global demand for low-cost energy generation over the last three decades, significant research efforts have been made towards the development and progress of organic solar cells (OSCs), in order to boost their competitiveness over silicon technology [1,2]. Owing to several attractive properties, including light weight, flexibility, low manufacturing costs, and compatibility with large-area processes, OSCs is considered as one of the most prominent photovoltaic technologies for sustainable energy production [3].

In this context, several polymeric donor:fullerene-based acceptor combinations have been flourished providing a rapid increase in the OSC devices efficiency over 9% [4,5,6,7]. On top of that, very recently, alternative optimized architectures such as tandem structures, novel donors and non-fullerene acceptor design and synthesis, as well as ternary systems have escalated the performance of OSCs over 14% [8,9,10,11].

Unlike to the typical binary OSC configuration, that is based on a donor–acceptor bulk heterojunction (BHJ) blend, the ternary one contains a third component which can function as: (i) second donor, (ii) second acceptor, and (iii) non-volatile additive [12]. The operation of a ternary OSC device relies on one of the four existing dominant mechanisms including: (1) charge transfer, (2) Forster resonance energy transfer, (3) parallel-linkage, and (4) alloyed donor structure mechanism [12,13]. Thus, according to the above mechanisms, small molecules [14,15,16,17,18], polymers [19,20,21], dye molecules [22,23], graphene-based materials [24,25] or 2D materials [12,26,27] could be chosen and incorporated as additives within the binary active layer.

This study discusses for the first time the design and synthesis of a novel graphene-based material (GO-TNF) through simple chemical processes as well as its direct incorporation in ink form within the binary active layer (PTB7:PC_71_BM) for the realization of inverted ternary OSC devices. GO-TNF consists of graphene oxide (GO) as core and TNF side groups linked with ethylenediamine (EDA) aliphatic spacers. Since the energy levels of the synthesized graphene-based molecule and these of PTB7 and PC_71_BM perfectly match, GO-TNF ink was incorporated in different ratios ranging from 1% to 3%. Upon the incorporation of GO-TNF, charge transfer operational mechanism dominated (cascade effect), while the photovoltaic performance was boosted in all ternary devices compared to the reference cell. The champion device, containing 2% *v*/*v* GO-TNF ink, exhibited a significant enhancement by ~13%, leading to a power conversion efficiency (PCE) of 8.71%. This increase is mainly due to the improvement of the nanomorphology between the donor:acceptor blend’s interfaces, as well as to electron mobility enhancement.

## 2. Materials and Methods

### 2.1. Materials

Initially, 9-oxo-fluorene-4-carboxylic acid 97%, 1,2-ethylenediamine (EDA) puriss. p.a., absolute, ≥99.5% (GC), graphite synthetic, H_2_SO_4_ 95–97%, fuming HNO_3_ 70%, SOCl_2_ ReagentPlus >99%, were purchased from Sigma Aldrich (Taufkirchen, Germany). PTB7 was purchased from Solaris Chem (Vaudreuil-Dorion, QC, Canada), while PC_71_BM and PFN were both purchased from Solenne BV (Groningen, The Netherlands). Finally, MoO_3_ and Al were bought from Kurt J. Lesker (East Sussex, UK), while the glass-ITO substrates were purchased from Naranjo Substrates (Groningen, The Netherlands).

### 2.2. Materials’ Synthetic Procedures

**Figure 1:**
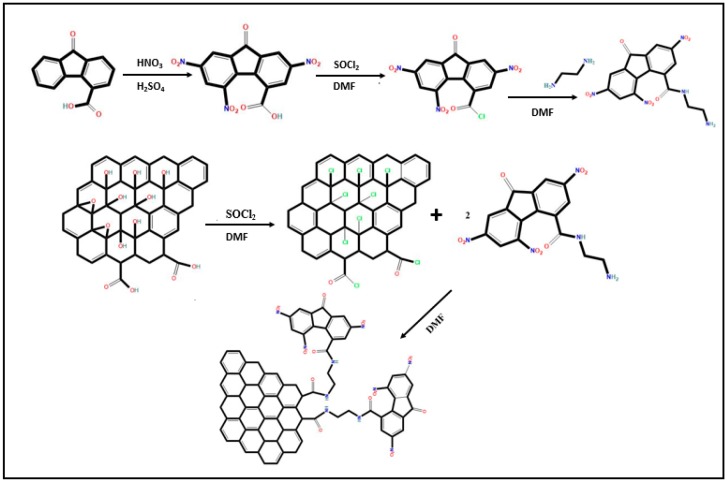
Schematic representation of the chemical synthetic procedure.

The preparation of GO-TNF took place into several steps, as it is depicted in Figure 1 and analyzed in Appendix A. First, 9-oxo-fluorene-4-carboxylic acid was nitrated using a mixture of concentrated sulfuric acid (H_2_SO_4_, 95–97%) and fuming nitric acid (HNO_3_, 70%), yielding 2,5,7-trinitro-9-oxo-fluorene-4-carboxylic acid (TNF-COOH). Afterwards, the carboxyl group of TNF was chlorinated using thionyl-chloride (SOCl_2_), to get 2,5,7-trinitro-9-oxo-fluorene-4-acyl-chloride (TNF-COCl). The linkage of TNF-COCl with 1,4-ethylenediamine (EDA) was held via a typical nucleophilic substitution reaction to obtain TNF-EDA. In the second parallel step, GO was prepared via a modified Hummers’ method [28] and was subsequently acylated, using SOCl_2_ to get GO-COCl [25]. The final GO-TNF was extracted upon the coupling between GO-COCl and TNF-EDA through a nucleophilic substitution reaction. Finally, GO-TNF ink was prepared as described in the SI (see Appendix A).

### 2.3. OSC Device Fabrication

**Figure 2:**
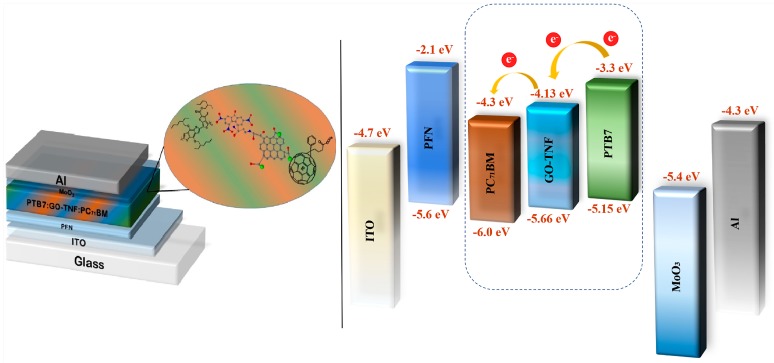
Schematic representation of the ternary OSC device (**left**) and energy levels diagram (**right**).

All OSC devices were fabricated in a typical sandwich inverted geometry consisting of a bottom indium tin oxide (ITO) coated glass substrates electrode, poly [(9,9-bis(3′-(N,N-dimethy-lamino)propyl)-2,7-fluorene)-alt-2,7-(9,9–dioctylfluorene)] (PFN) as the ETL, a PTB7:PC_71_BM BHJ thin film as the active layer, a MoO_3_ as the HTL and a top metal (Al) electrode. GO-TNF ink was directly incorporated within the binary photoactive layer, in ratios ranging from 1 to 3% ratio to the polymer for the fabrication of the ternary devices. The devices’ fabrication in detail is reported in the SI. The schematic representation of the device and the respective energy level diagram are depicted in Figure 2.

### 2.4. Characterization Techniques

ATR FT-IR (transmittance) experiments were carried out with a Bruker Vertex 70v FT-IR vacuum spectrometer equipped with a A225/Q Platinum ATR unit with single reflection diamond crystal which allows the infrared analysis of unevenly shaped solid samples and liquids through total reflection measurements, in a spectral range of 4000–700 cm^−1^. UV-vis absorption spectra were taken using a Shimadzu UV-2401 PC spectrophotometer, over the wavelength range of 270–800 nm. The photoluminescence (PL) measurements of the devices’ active layers were carried out at room temperature and resolved by using a UV grating and a sensitive, calibrated and liquid N_2_-cooled CCD camera in the wavelength range from 600 to 950 nm. The excitation source employed was a He-Cd CW laser at 325 nm with a full power of *P*_0_ = 35 mW. Raman measurements were performed at room temperature using a Horiba LabRAM HR Evolution confocal micro-spectrometer, in backscattering geometry (180°), equipped with an air-cooled solid-state laser operating at 532 nm with 100 mW output power. The laser beam was focused on the samples using a 10× Olympus microscope objective (numerical aperture of 0.25), providing a ~55 mW power on each sample. XRD patterns were collected on a Panalytical Expert Pro X-ray diffractometer, using Cu K_α_ radiation (λ = 1.5406 Å). Thermogravimetric analysis (TGA) was performed on 5–10 mg samples over the temperature range from 40 to 800 °C at a heating rate of 10 °C/min utilizing a Perkin-Elmer Diamond Pyris model under N_2_ atmosphere. The morphology of the surfaces was examined with an Atomic Force Microscope (Park Systems XE7, Park Systems Corporate Headquarters, Suwon, Korea). SEM images were taken through a JEOL JSM-7000F field emission scanning electron microscope. Cyclic voltammetry measurements were conducted on an Autolab PGSTAT302N. The photovoltaic performance of the devices was evaluated at room temperature within glove box (MBRAUN) conditions ((O_2_ < 0.1 ppm), moisture-free (H_2_O < 0.1 ppm)), and under standard illumination conditions with an Air Mass 1.5 Global (A.M. 1.5 G) solar simulator at an intensity of 1000 Wm^−2^ using an Agilent B1500A Semiconductor Device Analyzer, calibrated through a reference monocrystalline silicon solar cell supplied by Newport Corporation. The external quantum efficiency (EQE) measurements were conducted immediately after device fabrication, using an integrated system (Enlitech, Taiwan) and a lock-in amplifier with a current preamplifier, under short-circuit conditions. To enhance the credibility of our measurements, the solar simulator (a Xenon lamp) spectrum was calibrated using a monocrystalline photodetector of known spectral response. OSC devices were measured using a Xe lamp and an optical chopper at low frequencies (~200 Hz) in order to maximize the sound/noise (*S*/*N*) ratio. At least ten identical devices with six photovoltaics cells each were fabricated so that the reproducibility of the *J-V* characteristics is ensured.

## 3. Results and Discussions

### 3.1. ATR FT-IR Spectroscopy

**Figure 3:**
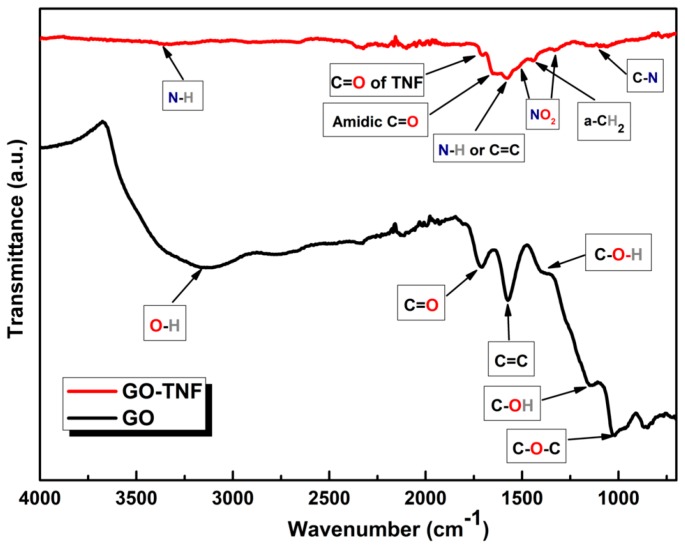
ATR FT-IR spectra of graphene oxide (GO) (black line) and GO-TNF (red line) in transmission mode.

ATR FT-IR spectra of GO and GO-TNF, in powder form, are presented in Figure 3. Pristine GO (black line) shows a broad and strong peak at 3390 cm^−1^, which is attributed to O-H stretching vibration of the OH- moieties. Furthermore, stretching vibration of C=O moieties is appeared at 1706 cm^−1^, while the remaining graphitic domains (C=C) stretching vibration are shown at 1568 cm^−1^. In addition, C-O-H bending vibration due to COOH groups are presented at 1394 cm^−1^. The peaks at 1143 cm^−1^ and 1027 cm^−1^ represent C-OH stretching vibration of the hydroxide domains and the stretching vibration of C-O-C groups, respectively. On the other hand, GO-TNF (red line), exhibits a broad peak of low intensity at 3331 cm^−1^, indicating a N-H stretching vibration. Next, a peak at 1697 cm^−1^ is attributed to C=O stretching vibration deriving from the carbonyl moiety of trinitrofluorenone. Moreover, two peaks occurred at 1652 cm^-1^ and 1575 cm^−1^ are due to amidic C=O stretching vibration. NO_2_ asymmetric and symmetric stretch vibrations are shown at 1525 cm^−1^ and 1334 cm^−1^, respectively, as well as the peak at 1446 cm^−1^ corresponds to the aliphatic a-CH_2_ bending vibration of ethylenediamine moiety. Finally, C-N stretching vibration of the ethylene diamine moieties appears at 1105 cm^−1^. ATR FT-IR spectra of the intermediate TNF and EDA-TNF are reported in the SI.

### 3.2. UV-Visible Measurements

**Figure 4:**
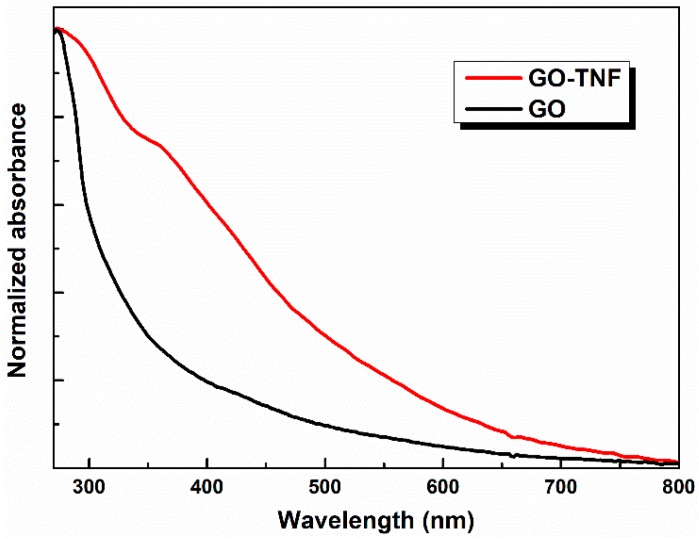
UV-vis spectra of GO (black line) and GO-TNF (red line).

In Figure 4, UV-vis spectra of GO and GO-TNF in solid state are presented. Due to the strong attachment of TNF moieties to the edges of the lattice of GO, the absorption spectrum of GO-TNF is broader than that of the pristine GO, exhibiting a shoulder at ~365 nm. This fact indicates that there is a strong interaction between the GO lattice and TNF moieties, which is mainly attributed to the enhanced electron delocalization caused by TNF [24].

### 3.3. Raman Spectroscopy

**Figure 5:**
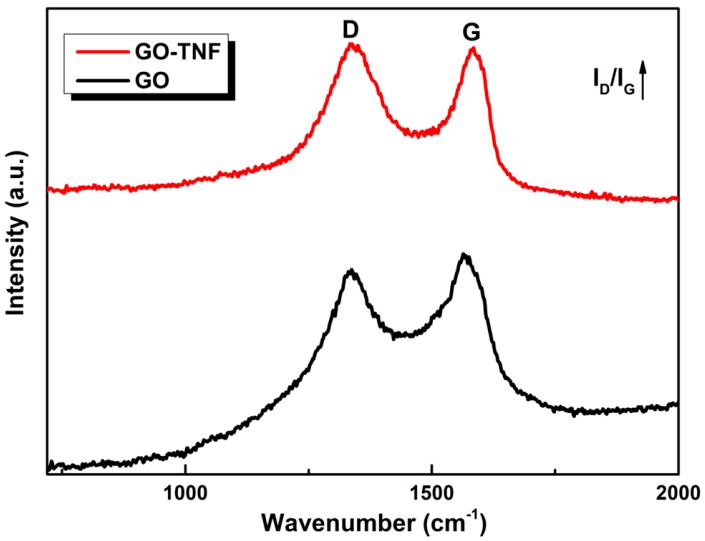
Raman spectra of GO and GO-TNF.

Raman spectra of GO and GO-TNF are shown in Figure 5. No shift is observed for both D and G peaks of GO compared to the respective ones of GO-TNF; D bands occurred at ~1340 cm^−1^, while G bands at ~1580 cm^−1^. However, a difference in the relative intensity ratio (I_D_/I_G_) was observed from 0.92 for GO to 1.04 for GO-TNF, indicating that the linking between GO and TNF increased disorder and defects in the graphitic lattice [25].

### 3.4. Photoluminescence (PL) Spectroscopy

**Figure 6:**
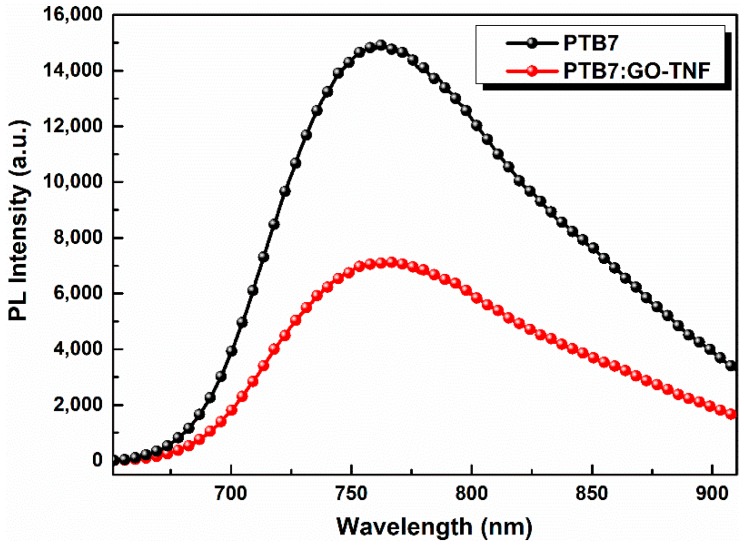
PL spectra of PTB7 (black) and PTB7:GO-TNF (2%) (red).

Photoluminescence (PL) measurements were conducted to evaluate charge transfer mechanism upon the incorporation of GO-TNF within the active layer, and the respective PL spectra are depicted in Figure 6. In this context, PTB7 and PTB7:GO-TNF thin films were excited at 471 nm presenting an emission band around 760 nm corresponding to radiative decay of photogenerated excitons from the excited state to ground state [29]. When 2% *v*/*v* GO-TNF ink was added, PL intensity quenching is significant owing to the better energy offset between the LUMO levels of PTB7 and GO-TNF that enhances the charge transfer mechanism. In our case, the incorporation of GO-TNF ink with an optimum concentration of 2% *v*/*v*, facilitates exciton dissociation at the PTB7:PC_71_BM interface thus leading to a higher number of electrons that can be collected by the cathode, which is in agreement with the champion current density value achieved so far (17.65 mA cm^−2^) [30].

### 3.5. XRD Measurements

**Figure 7:**
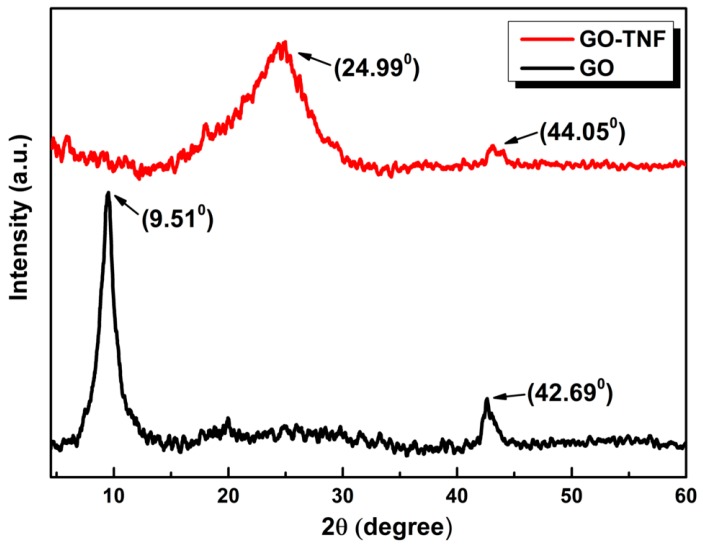
X-ray diffraction patterns of GO (black) and GO-TNF (red).

The crystallinity of pristine GO and GO-TNF was investigated by X-ray diffraction (XRD) in a 2θ range from 5° to 60° (Figure 7). GO displays a narrow peak at 9.51° which is attributed to the main reflection (002) of its stacks with an interlayer d-spacing of ~8.2 Å, while a second weak peak appearing at 42.69° is due to the turbostratic band of disordered carbon materials [31]. On the other hand, GO-TNF exhibits a broad peak at 24.99° referring to (002) reflection with a slightly increased d-spacing of 9.1 Å which is attributed to GO covalent bonding with TNF moieties.

### 3.6. Thermogravimetric (TGA) Analysis

**Figure 8:**
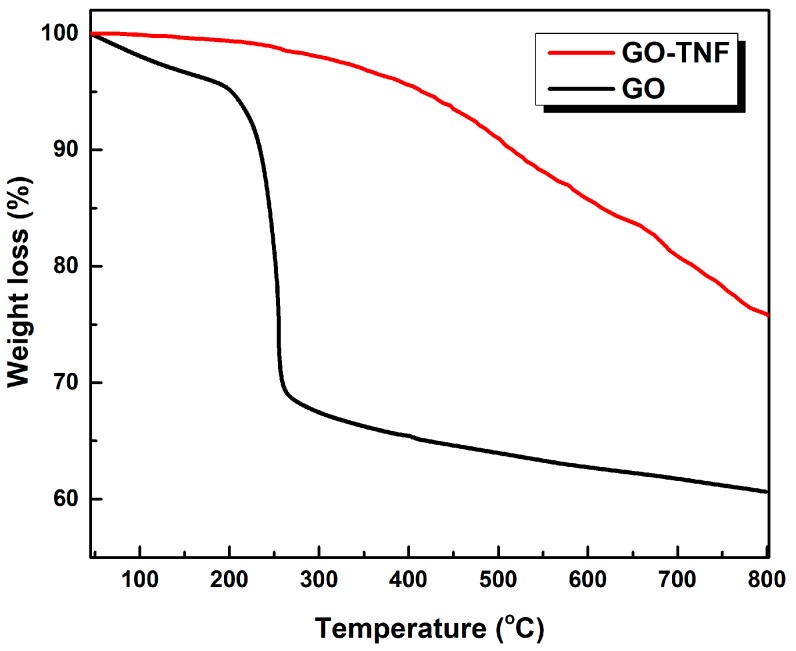
TGA curves of GO (black) and GO-TNF (red) taken under N_2_ atmosphere and 10 °C/min heating rate.

Figure 8 displays the TGA curves of GO and GO-TNF obtained under inert atmosphere with a heating rate of 10 °C/min, while the maximum temperature limit was set at 800 °C. First, GO exhibited a moderate weight loss of 5% at a 210 °C, which was followed by a steep weight loss of 37% at about 270 °C due to oxygen functional groups’ pyrolysis. Its total mass loss was 40% at 800 °C. On the other hand, GO-TNF presented an improved thermal stability when compared to GO, since the total loss did not exceed 22% of its initial weight. The improved thermal stability of GO-TNF was attributed to the successful amide bond formation between GO and TNF that enhances thermal stability [32].

### 3.7. Cyclic Voltammetry Measurements

**Figure 9:**
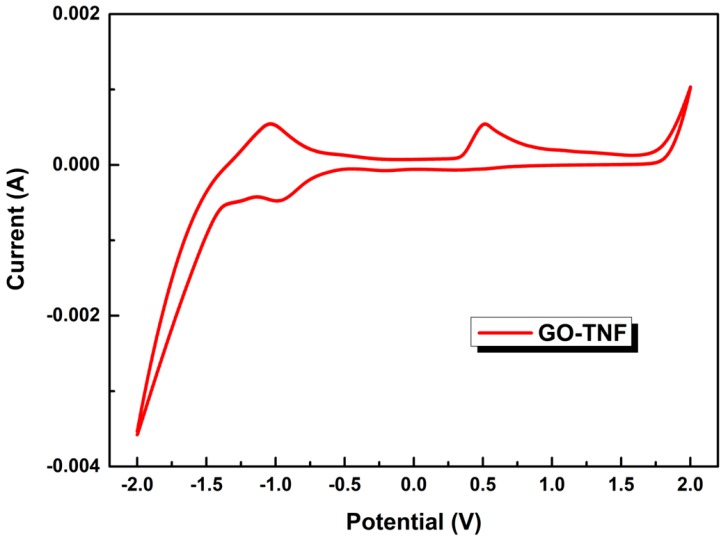
Cyclic voltammogram of GO-TNF.

To determine the energy levels of GO-TNF, cyclic voltammetry measurements were carried out using an electrolytic solution of TBAPF6 in CH_3_CN 0.1M, with a scan rate of 10 mVs^−1^, between the potential sweep window of −2 V to +2 V, as demonstrated in Figure 9. The energy HOMO and LUMO levels of GO-TNF were calculated using the empirical relations below [33]:

E_HOMO_ = −(E_(onset,ox vs Fc+)⁄Fc]_ + 5.1)(eV) (1)

E_LUMO_ = −(E_(onset,red vs Fc+)⁄Fc]_ + 5.1)(eV) (2)

The HOMO level was approximately −5.66 eV as calculated by the oxidation peak onset 0.53 V, while its LUMO level was extracted from the onset of the reduction peak (0.85 V) and was −4.13 eV.

### 3.8. Microscopic Characterization

The morphology of GO-TNF was examined using field emission scanning electron microscopy (FE-SEM). Representative SEM images of its flakes coated on silicon substrates are demonstrated in Figure S11. The size of the wrinkled GO-TNF flakes varies ranging from 100 nm to 1 μm, while it should be reported that no charging was observed during SEM imaging, thus indicating that the formed network was electrically conductive.

### 3.9. Photovoltaic Performance Evaluation

**Figure 10:**
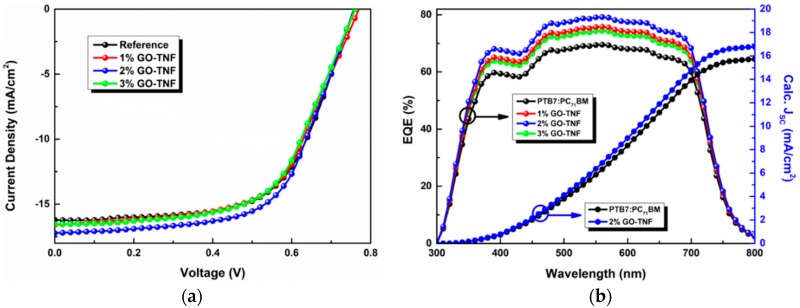
*J-V* characteristics (**a**) and EQE curves (**b**) of the reference (PTB7:PC_71_BM) and the devices incorporating different GO-TNF ink content. The calculated *J_sc_* curves (inset in EQE) correspond to the reference and the champion device with 2% GO-TNF ink content.

Several OSC devices were fabricated via the incorporation of GO-TNF ink within the binary PTB7:PC_71_BM photoactive layer (Figure 1) in various ratios (1%, 2%, and 3%) and *J-V* characteristic curves were exported to evaluate its operational role into device’s photovoltaic (PV) performance (Figure 10). It is obvious that the presence of GO-TNF was beneficial, as stated below in Table 1.

All ternary devices showed an improved performance, especially the device containing 2% *v*/*v* GO-TNF ink. In particular, the champion device exhibited a current density (*J_sc_*) of 17.65 mA/cm^2^ and a power conversion efficiency (PCE) of 8.71% that show an improvement of ~10% and ~13% respectively when compared to the reference device.

To further confirm the experimental *J_sc_* improvement due to the incorporation of GO-TNF, external quantum efficiency measurements were conducted to determine the calculated *J_sc_*. Figure 10 depicts the external quantum efficiency (EQE) curves of the reference, as well as the champion ternary OSC device incorporating 2% *v*/*v* GO-TNF ink.

It can be seen that the EQE enhancement is in full accordance to *J_sc_* increase, as calculated from the respective *J-V* curves depicted in Figure 10a. In addition, the absence of any new peak is in agreement with the operational role of GO-TNF, which does not contribute in exciton generation but only in electron transfer, hence confirming the charge transfer mechanism (cascade effect). The accuracy of the PV measurements was checked, by calculating the *J_sc_* values of the OSCs from the integration of the EQE spectra. The calculated *J_sc_* was found to be −15.72 mA cm^−2^ and −16.78 mA cm^−2^ for the binary and the 2% GO-TNF ink content ternary devices, respectively, which are within the standard deviation from the *J_sc_* obtained from the *J-V* curves. It should be also noted that any concentration of GO-TNF ink higher than 3% wt. resulted in a short circuit, probably due to the occurrence of local shunts. This undesired effect could be linked with the concentration of GO-TNF ink in the blend that becomes enough to allow a direct bridging with the ITO electrode.

To get a more accurate insight into the influence of GO-TNF blend into the charge transfer process in the ternary approach, hole-only and electron-only cells were fabricated to calculate the hole and electron mobility, respectively. Measurements were based on space charge limited current method. Hole-only cells and electron-only cells were fabricates using the architecture indium tin oxide ITO/PEDOT:PSS/PTB7:GO-TNF:PC_71_BM/MoO_3_/Al for holes and ITO/PFN/PCDTBT:GO-TNF:PC_71_BM/Ca/Al for electrons, respectively. The evaluation of the charge carrier mobilities was based on the Mott–Gurney equation [34]:

$$J_{SCLC}=\frac{9}{8}\varepsilon_{r}\varepsilon_{0}\mu \frac{{\left(V-V_{bi}\right)}^{2}}{d^{3}}$$

where *ε_r_* is the relative dielectric constant, *ε*_0_ is the permittivity of free space, *µ* is the charge carrier mobility, *V* is the applied voltage, *V_bi_* is the built-in potential, and *d* is the active layer thickness.

Supplementary Figure S12 illustrates *J-V*^2^ characteristics under dark conditions for (a) electron-only and (b) hole-only devices, respectively, where the black line refers to the control device (PTB7:PC_71_BM), while the red line corresponds to the champion ternary one PTB7:GO-TNF (2%): PC_71_BM. According to *J-V*^2^ characteristics, although hole mobility did not present any significant change upon the addition of GO-TNF, the respective electron mobility has shown a significant improvement, passing from 7.80 × 10^−5^ cm^2^ V^−1^ s^−1^ to 9.93 × 10^−5^ cm^2^ V^−1^ s^−1^ (Table 2). This improvement in electron mobility obviously originates from the presence of GO-TNF which has favourable energy levels, located between the energy levels of the donor and acceptor materials, respectively. Hence, the observed electrons’ mobility enhancement is directly associated with the cascade effect facilitating electrons’ transition from PTB7 to the ITO electrode [35].

In Appendix A, supplementary Figure S13 represents a simple one diode equivalent circuit model corresponding to the ternary OSC device incorporating 2% GO-TNF ink. The diode “D” corresponds to the electrical equivalent of the optical losses at the surface of bulk heterojunction. “R_sh_” represents the leakage and recombination losses whereas “R_S_” represents the sum of the internal resistance, including the resistance of the active layer and ohmic contact.

Due to photoluminescence, the generated excitons are diffused to the nearest D: A interface and are dissociated to form polaron-pairs. Polaron pairs can be either recombined or dissociated into free carriers and are subsequently extracted to the electrodes through a cascade-diffusion process. GO-TNF plays a key role in this cascade process, facilitating charge transfer from the polymer to the fullerene reducing the possibility of free carriers’ recombination. This can be proven, by calculating the overall resistance at the maximum power point which represents the decrease of the overall resistance that derives from the higher recombination rate, resulting in larger R_sh_ value (Supplementary Figure S14).

### 3.10. Morphology Characterization of the Active Layer

**Figure 11:**
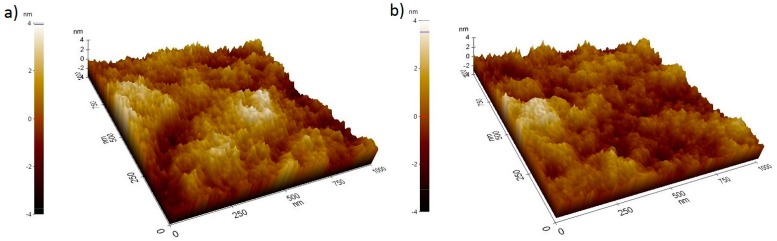
AFM images of (**a**) the reference PTB7:PC_71_BM active layer and (**b**) the champion ternary device containing 2% of GO-TNF ink.

The morphology of the reference PTB7:PC_71_BM as well as the champion ternary blend (2%) surfaces were both examined by atomic force microscopy (AFM), with a scan size of 1 mm by 1 mm, as displayed in Figure 11. The said ternary active layer exhibited a slightly smoother surface than the binary one, giving a root-mean-square (RMS) roughness of 1.15 nm and 1.22 nm, respectively. The fact that the morphology was improved upon the incorporation of 2% GO-TNF is in full accordance to *J_sc_* increase, indicating that the possibility of energetic disorders formation is smaller in case of the ternary device. On the other hand, *V_oc_* slightly changed upon the addition of GO-TNF, which is normal, since GO-TNF was incorporated in very low concentrations that cannot significantly affect *V_oc_* values.

## 4. Conclusions

Herein, we reported the synthesis and characterization of a new graphene-trinitrofluorenone derivative, named GO-TNF, as well as its operational role as the cascade material between the LUMO energy levels of PTB7 and PC_71_BM, in efficient ternary OSCs. Due to its ideal energy levels, it was directly incorporated, as the third component, within the binary photoactive layer providing a significant improvement in current density of the champion device (2% *v*/*v* GO-TNF ink) by ~10%. Respectively, the PCE value of the same device was higher by ~13%, leading to a champion efficiency of 8.71%. Our efforts proved that the rational design of graphene-based molecules could provide the opportunity for novel materials synthesis with tunable properties to be incorporated as additives, even as interlayers into organic, as well as hybrid solar cells, thus contributing to the realization of new generation high performance PV systems.

## Figures and Tables

**Table 1:** Photovoltaic characteristics summary of the OSC devices based on PTB7:GO-TNF:PC_71_BM ternary blends ^a^.

GO-TNF Content (%)	*J_sc_* (mA/cm^2^)	Calc. *J_sc_* (mA/cm^2^)	V_oc_ (V)	FF (%)	PCE (%)
**0**	16.20 ± 0.45	15.72	0.760 ± 0.010	61.8 ± 0.7	7.61 ± 0.11
**1**	16.54 ± 0.54	16.21	0.760 ± 0.005	63.0 ± 0.4	7.92 ± 0.26
**2**	17.21 ± 0.44	16.78	0.760 ± 0.011	64.0 ± 0.1	8.37 ± 0.34
**3**	16.53 ± 0.35	16.21	0.760 ± 0.009	62.4 ± 0.6	7.84 ± 0.17

^a^ The data is averaged from 10 identical devices with 6 cells each.

**Table 2:** Hole and electron mobilities of PTB7:PC_71_BM and ternary blend PTB7:GO-TNF:PC_71_BM *.

Active Layer	μ_h_ (cm^2^ V^−1^ s^−1^)	μ_e_ (cm^2^ V^−1^ s^−1^)	Ratio (μ_h_/μ_e_)
**PTB7:PC_71_BM** (reference)	1.28 × 10^−4^	7.80 × 10^−5^	1.64
**1% GO-TNF**	1.31 × 10^−4^	8.71 × 10^−5^	1.50
**2% GO-TNF**	1.39 × 10^−4^	9.93 × 10^−5^	1.39
**3% GO-TNF**	1.34 × 10^−4^	8.03 × 10^−5^	1.67

* The data were averaged from 10 identical devices with 6 cells each.

## Supplementary Materials

The following are available online at https://www.mdpi.com/2079-4991/10/1/89/s1, Figure S1: The reaction mechanism of the nitration of 9-oxo-fluorene-4-carboxylic acid, Figure S2: The reaction mechanism of acyl chloride synthesis, Figure S3: The reaction mechanism of the amide bond formation, Figure S4: Schematic of 9-oxo-4-carboxyl-fluorenone nitration, Figure S5: ATR FT-IR spectrum of TNF-COOH, Figure S6: Schematic representation of TNF-COCl synthesis, Figure S7: The reaction representation of TNF-EDA synthesis, Figure S8: ATR FT-IR spectrum of TNF-EDA, Figure S9: The reaction representation of GO-COCl preparation, Figure S10: (i) Schematic of the final reaction of GO-TNF, (ii) GO-COCl and TNF-EDA in powder form (a), setup of the reaction (b), GO-TNF in powder form (c), Figure S11: FE-SEM images of GO-TNF, Figure S12: *J-V*^2^ characteristics of the fabricated (a) electron-only and (b) hole-only devices, Figure S13: The one diode equivalent circuit model corresponding to the ternary OSC device incorporating 2% GO-TNF ink, Figure S14: The effect of shunt resistance.

## Appendix A

The synthetic procedure of GO-TNF and its intermediate stages, as well as explanations of experimental details to understanding and reproducing the research are available.
