# Supplementary material for: Emphasizing the Operational Role of a Novel Graphene-Based Ink into High Performance Ternary Organic Solar Cells
Source: Nanomaterials (Basel). 2020 Jan 2;10(1):89. doi: 10.3390/nano10010089 (PMC7023384; doi:10.3390/nano10010089)
Supplement: Supplementary file 1 [file nanomaterials-10-00089-s001.pdf]

nanomaterials

Article

Emphasizing the Operational Role of a Novel Graphene-based Ink into High Performance Ternary Organic Solar Cells

Minas M. Stylianakis [1,*,†], Dimitrios M. Kosmidis [1,†], Katerina Anagnostou [1], Christos Polyzoidis [1], Miron Krassas [1,2], George Kenanakis [3], George Viskadouros [1,4], Nikolaos Kornilios [1], Konstantinos Petridis [1,5] and Emmanuel Kymakis [1,*]

1 Department of Electrical & Computer Engineering, Hellenic Mediterranean University (HMU), Estavromenos, Heraklion 71410, Greece; kosdimitris@hmu.gr (D.M.K); katerinanag@hmu.gr (K.A.); polyzoidis@hmu.gr (C.P.); kmiron@hmu.gr (K.M.); viskadouros@hmu.gr (G.V.); kornil@hmu.gr (N.K.)
2 Department of Materials Science and Technology, University of Crete, Heraklion 71003, Crete, Greece
3 Institute of Electronic Structure and Laser, Foundation for Research and Technology-Hellas, N. Plastira 100, Heraklion, 70013 Crete, Greece; gkenanak@iesl.forth.gr (G.K.)
4 Department of Mineral Resources Engineering, Technical University of Crete, Chania 73100 Crete, Greece
5 Department of Electronic Engineering, Hellenic Mediterranean University (HMU), Chania 73132, Greece; c.petridischania@gmail.com (C.P.)
† These authors contributed equally to this work.
* Correspondence: stylianakis@hmu.gr (M.M.S.); kymakis@hmu.gr (E.K.); Tel.: +30-2810-379775 (M.M.S.)

Received: date; Accepted: date; Published: date

Supporting Information

Experimental

1. Reaction Mechanisms

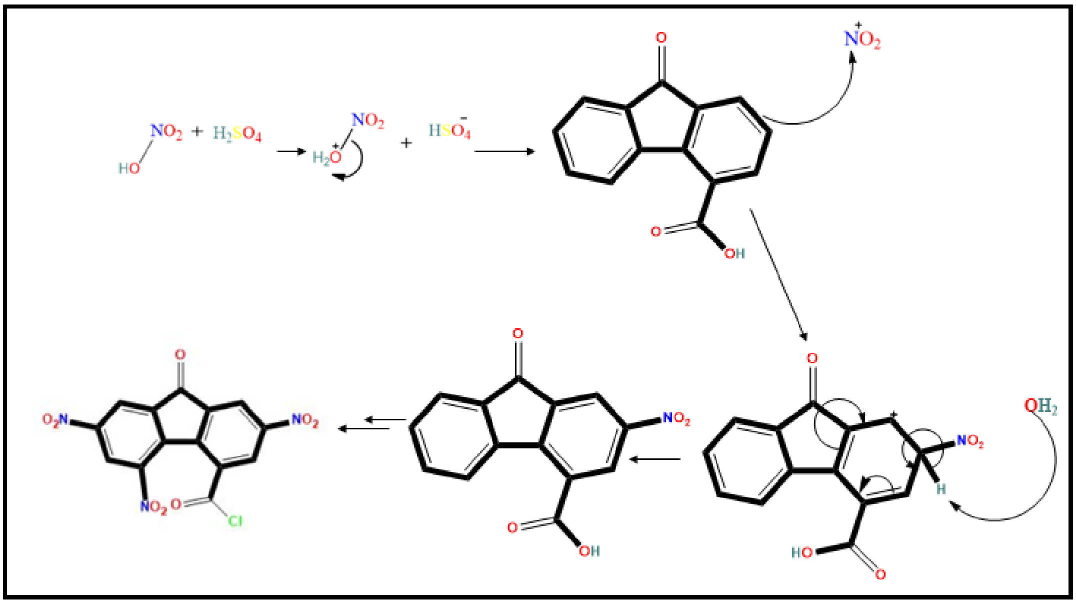

Figure S1. The reaction mechanism of the nitration of 9-oxo-fluorene-4-carboxylic acid.

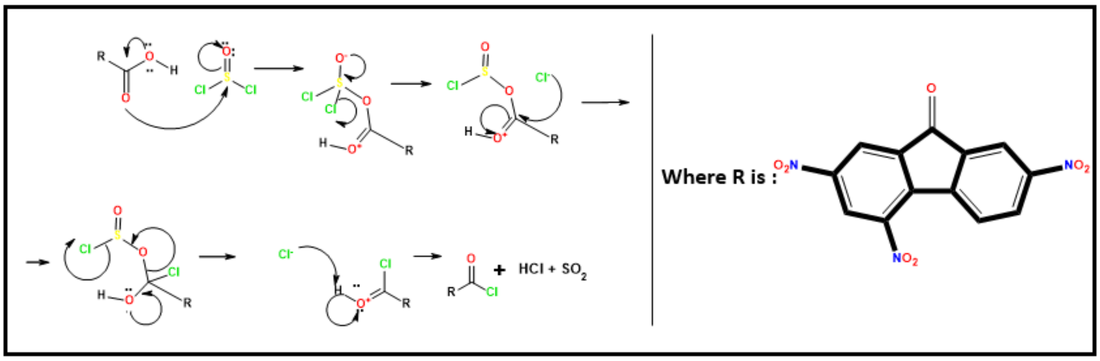

Figure S2. The reaction mechanism of acyl chloride synthesis.

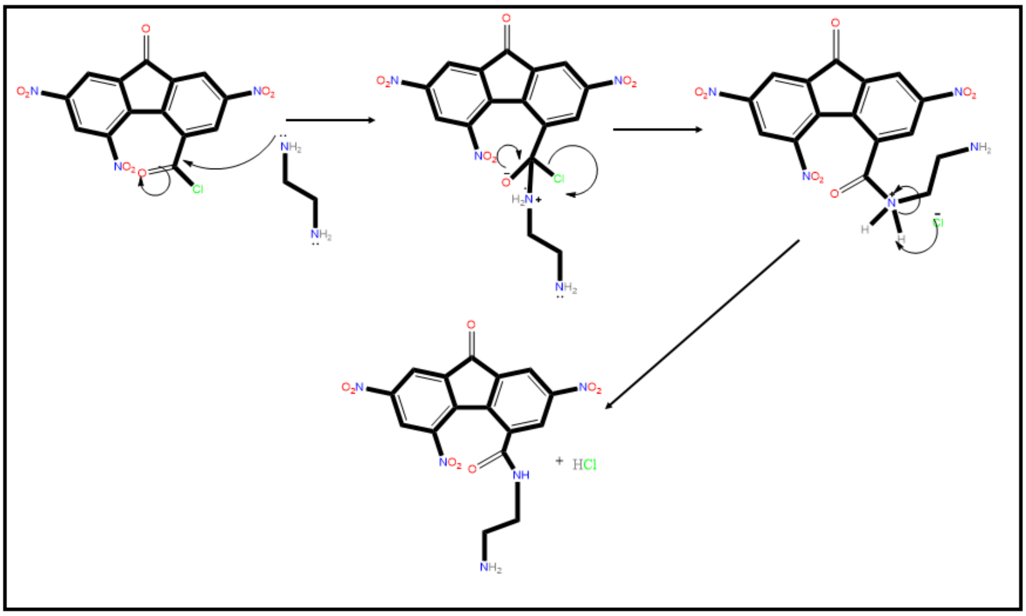

Figure S3. The reaction mechanism of the amide bond formation.

2. Materials Synthesis

2.1 Preparation of TNF-COOH

8 ml of concentrated (95-97 %) sulfuric acid (H_2SO_4) were added slowly to a separation funnel containing 1 g of 9-oxo-fluorene-4-carboxylic acid. The color of H_2SO_4 changed immediately after the addition from colorless to dark red. The solution was firmly mixed and added dropwise, over a 15-minute period of time, to a refluxing mixture of 13 ml fuming nitric acid (HNO_3) and 8 ml of concentrated H_2SO_4. The temperature of the refluxing mixture of concentrated H_2SO_4 and fuming HNO_3 was 85 ^{o}C. Afterwards, a mixture of 9 ml fuming HNO_3 and 11 ml of concentrated H_2SO_4 was added dropwise over a 4.5 hours period of time. After cooling to room temperature, the reaction mixture was left under N_2 atmosphere and stirring overnight. Subsequently, the reaction solution was poured into ice water (100 mL) and the yellow solid which precipitated was collected onto a G5 filtration funnel. The yellow solid was washed with 5 ml of 0.05 % aqueous solution of sodium bicarbonate in order to remove any amount of residual acid. Then it was dried overnight at 40 ^{o}C. Afterwards, it was collected and recrystallized with methanol. The recrystallization process occurred with the dissolution of the product to the smallest possible amount (5-7 ml) of methanol that was brought to reflux, followed by the placement of the solution flask to the refrigerator overnight. The residual precipitate was then collected on a G5 filtration funnel and dried at 40 ^{o}C overnight. TNF-COOH in powder form was characterized by ATR FT-IR (**Figure S5**).

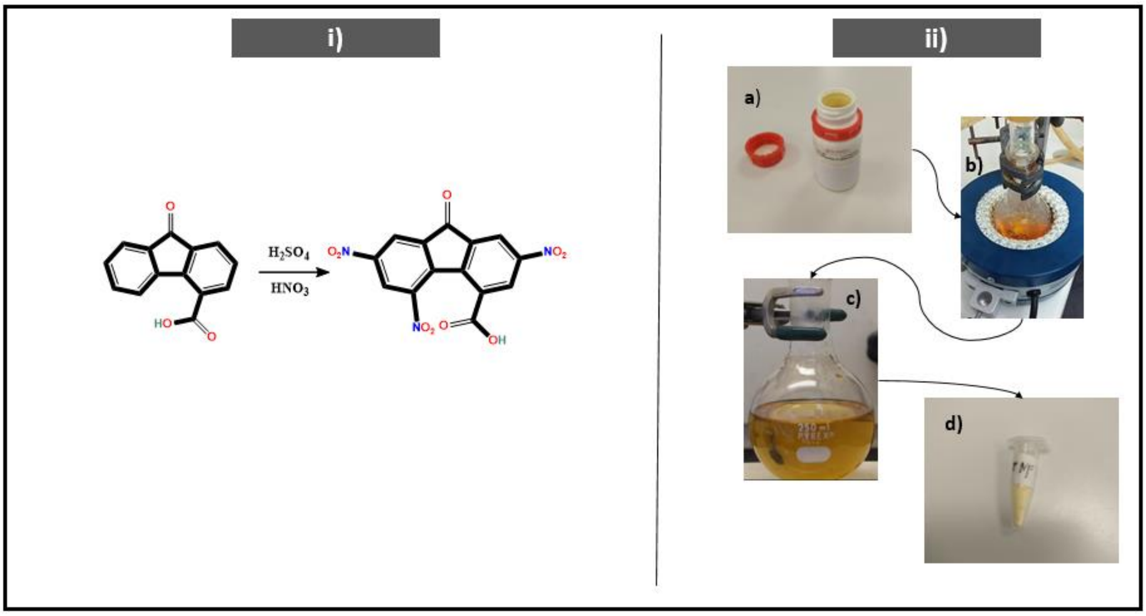

Figure S4. Schematic of 9-oxo-4-carboxyl-fluorenone nitration. ii) powder of 9-oxo-4-carboxyl-fluorenone (a), the reaction setup (b), the reaction solution (c), the final product (d).

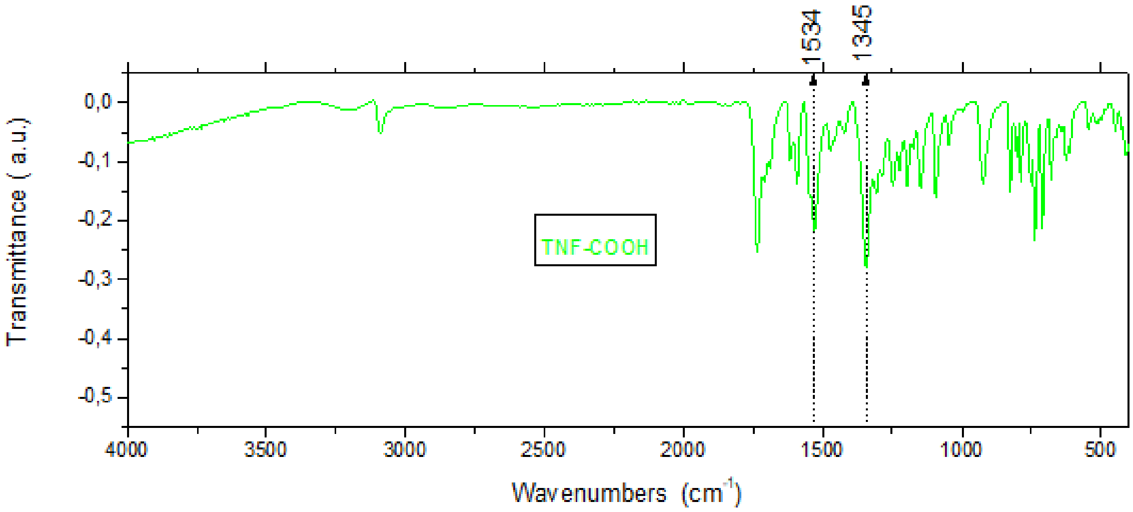

Figure S5. ATR FT-IR spectrum of TNF-COOH. The peaks at 1534 and 1345 cm^{-1} are attributed to NO_2 stretching vibrations, which strongly indicate the successful nitration of 9-oxo-fluorene-4-carboxylic acid.

2.2 Preparation of 2,5,7-trinitro-9-oxo-fluorene-4-acyl-chloride (TNF-COCl)

0.5 g of TNF, 25 ml of thionyl chloride ($SOCl_2$) and 1 ml of DMF were added to a 50 mL round bottom flask. Subsequently, the reaction solution was brought to reflux condition (75 °C) under N_2 atmosphere for 18 hours. Afterwards, $SOCl_2$ was removed via distillation at 85 °C and the residual precipitate was washed with diethyl-ether, in order to completely remove any amount of $SOCl_2$ that remained after the distillation process was completed. Finally, the product of TNF-COCl was recrystallized with a very small amount of toluene and dried overnight at 60 °C. The product was used immediately after its preparation in order to avoid degradation.

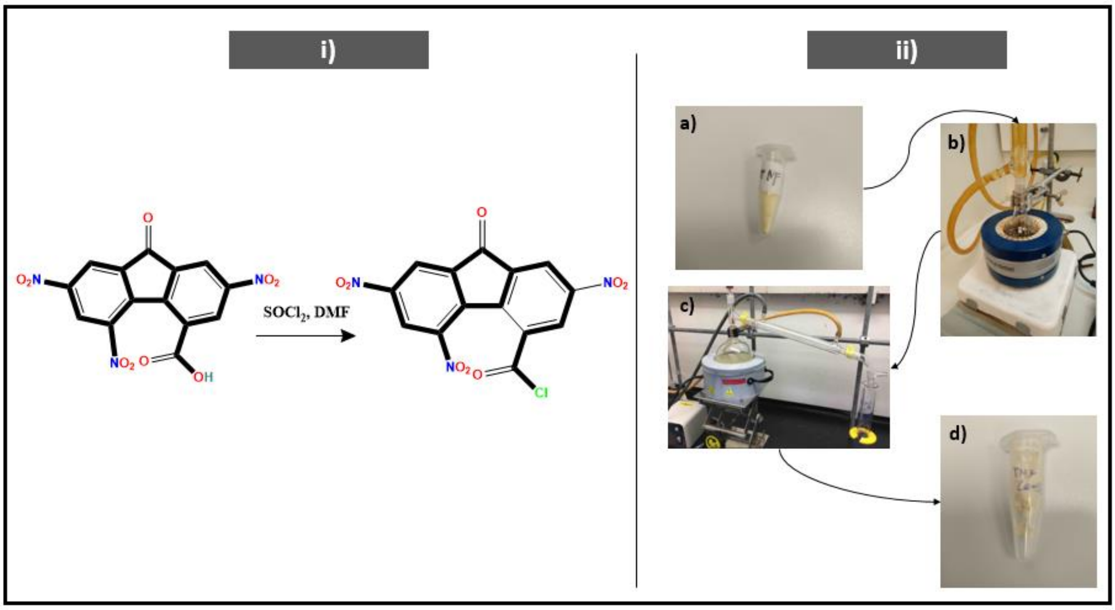

Figure S6. Schematic representation of TNF-COCl synthesis. ii) TNF in powder form (a), the reaction setup b), the distillation setup (c), TNF-COCl in powder form (d).

2.3 Preparation of TNF-EDA

0.3 g of TNF-COCl and 12 ml of THF were added in a dried 50 ml round bottom flask. Then, the reaction mixture was placed in an ice bath until the temperature dropped to 3 °C. Subsequently, 0.2 ml of ethylenediamine (EDA) was added under continuous stirring (**Figure S7**). A sharp color change was observed after the addition from yellowish to light brownish. During the next hour, 1 ml of triethylamine was added dropwise, in order to adduct the producing HCl, precipitate as a salt and increase the reaction yield. The reaction mixture was centrifuged, and the residual precipitate was washed with slightly acidified water. Thereupon, several washes with 2D water of the solid were conducted and the product was placed in the oven at 60 °C for 6 h to dry. TNF-EDA in powder form was characterized by ATR FT-IR (**Figure S8**).

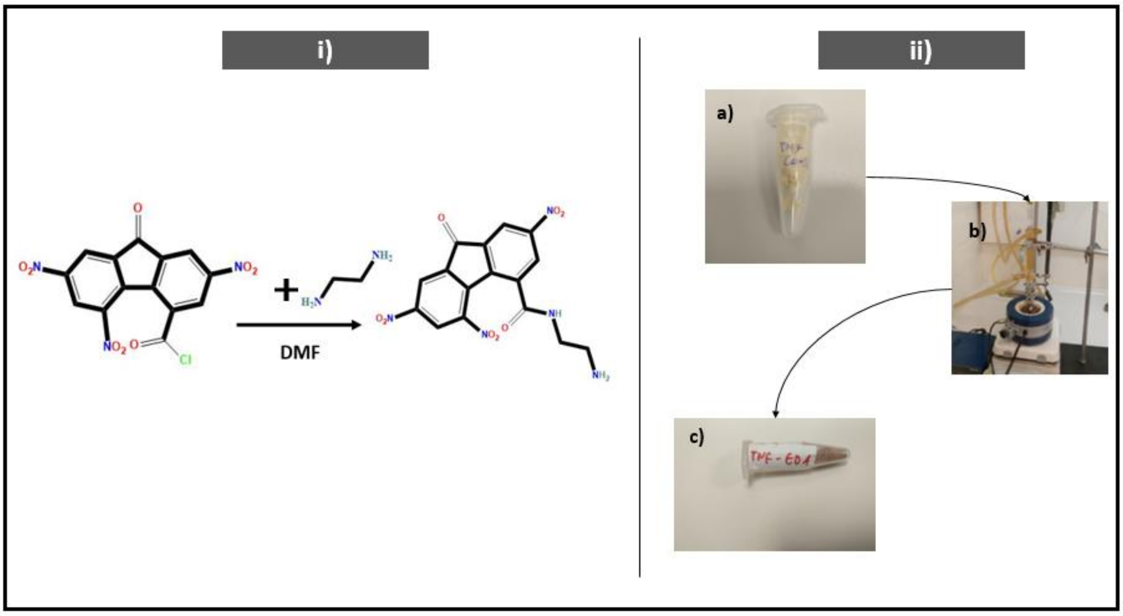

Figure S7. The reaction representation of TNF-EDA synthesis. ii) TNF-COCl powder (a), the reaction setup (b), TNF-EDA in powder form (c).

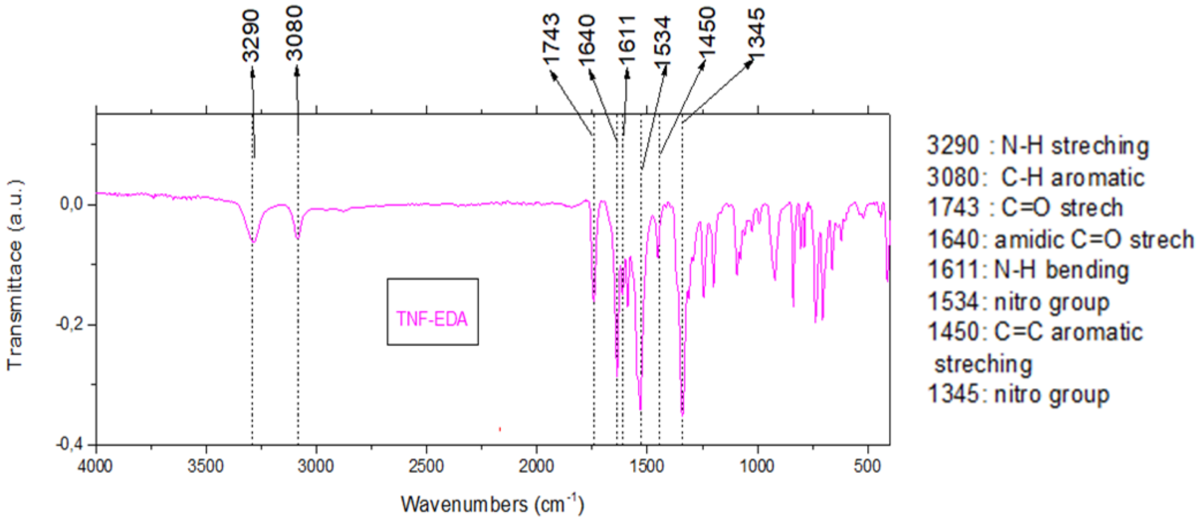

Figure S8. ATR FT-IR spectrum of TNF-EDA.

2.4 Preparation of Graphene Oxide (GO)

GO was synthesized from graphite powder according to the modified Hummer's method. 1 g of graphite was placed in a 400 ml reaction beaker. Then the beaker was placed in an ice bath and 40 ml of concentrated H_2SO_4 were added. The mixture was left under stirring for 20 minutes and 1 g of $NaNO_3$ was added slowly while the ice from the ice bath was being frequently replaced. After another hour of stirring in low temperature, 6 g of $KMnO_4$ were added very slowly to the reaction mixture. The solution was left under stirring overnight and then it was heated for 100 minutes at 35 °C. Afterwards, the mixture was heated to 90 °C and 80 ml of water was added. Continuously, it was left under stirring for another 40 minutes. Thereupon, the heating was turned off and 200 ml of water were added very slowly. Subsequently, 20 ml of H_2O_2 were added very slowly in order to react with the residual $KMnO_4$. The reaction mixture was left to cool down to room temperature and then was centrifuged. The residual precipitate was washed with 200 ml of hot water (65 °C). Several washes with water (25 °C) followed until the pH of the supernatant was 7. The precipitate was dried for 5 days at 80 °C. Afterwards, it was pulverized and stored in vials for further use.

2.5 Acylation of GO, preparation of graphene oxide acyl-chloride (GO-COCl)

30 mg of GO, 20 ml $SOCl_2$ and 0.5 ml of DMF were added in a dried 100 ml round bottom flask. The mixture was sonicated for 1 hour and was left under N_2 atmosphere in reflux condition for 24 h. Afterwards, the solvent was removed via distillation and the resulting precipitate was washed with THF multiple times. Then, the product was dried in an oven overnight at 60 °C. The reaction process is demonstrated in **Figure S9**.

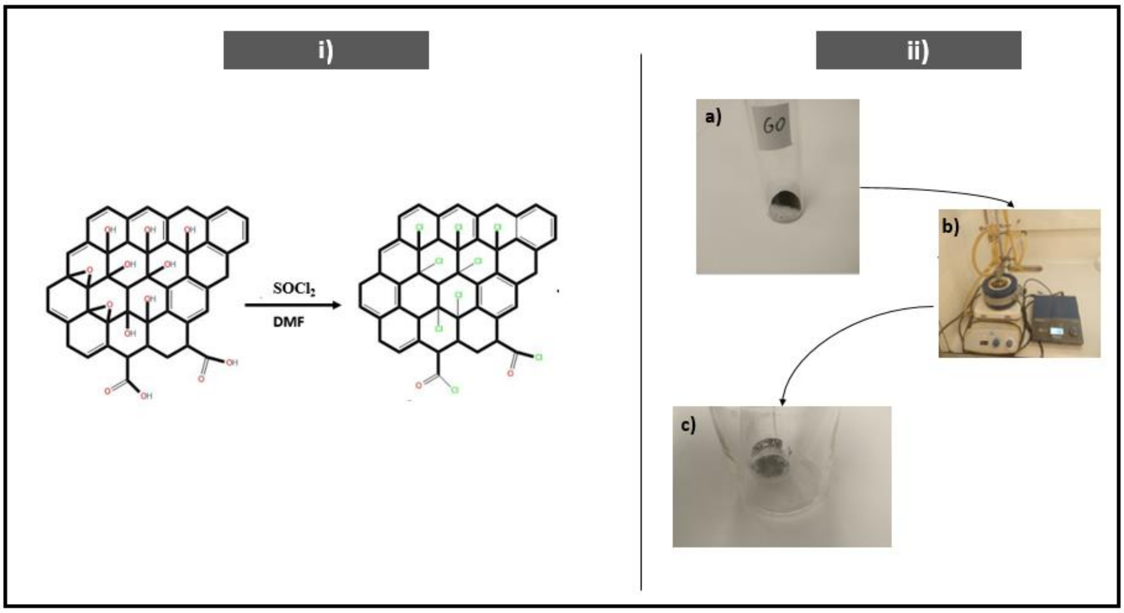

Figure S9. i) The reaction representation of GO-COCl preparation. ii) GO in powder form (a), the reaction setup (b), the yielded GO-COCl (c).

2.6 Synthetic procedure of the final GO-TNF

13 mg GO-COCl and 42.4 mg of TNF-EDA were dispersed in 15 ml of dimethylformamide by a 10-minute ultrasonication at room temperature. Subsequently, 2 ml of triethylamine were added to the reaction flask. The mixture was stirred and refluxed (137 °C) for 72h, under N_2 atmosphere. Afterwards, the mixture was cooled at room temperature and poured in a centrifugal funnel, along with the solid product that was collected from the walls of the reaction flask. The reaction solution was centrifuged for 1.5 h and the solid was collected and washed with 2-4 ml of methanol (primary product). The supernatant was mixed with 15 ml of anhydrous diethyl-ether in order to boost the precipitation of the secondary product. Then, the residual precipitate was centrifuged for 100 minutes, washed with ethanol 5 times and put to the oven at 45 °C overnight.

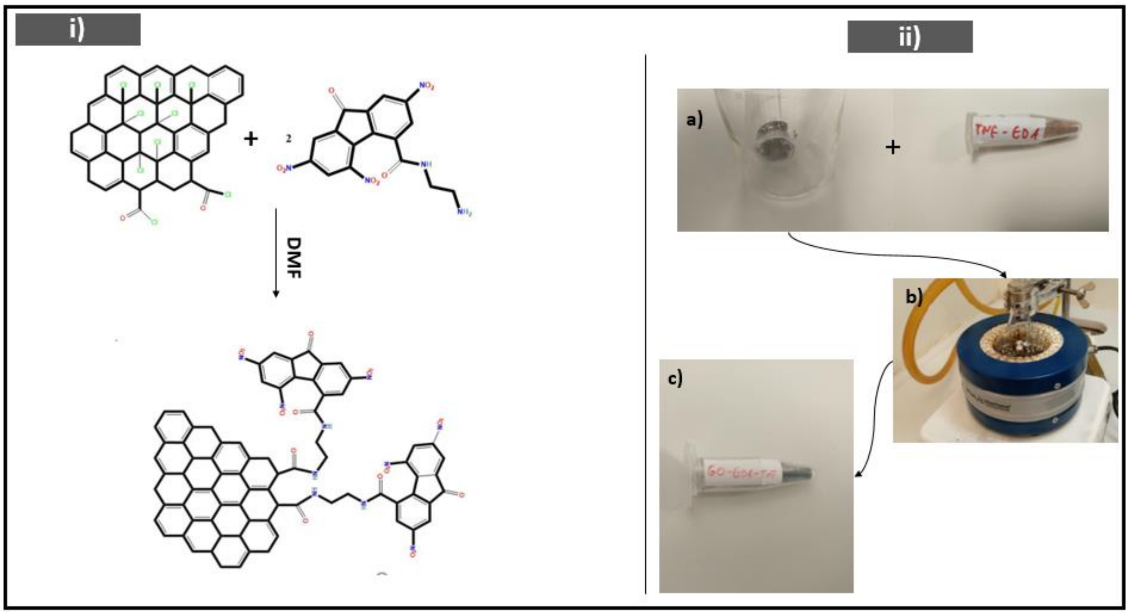

Figure S10. i) Schematic of the final reaction of GO-TNF. ii) GO-COCl and TNF-EDA in powder form (a), setup of the reaction (b), GO-TNF in powder form (c).

2.7 GO-TNF ink preparation

GO-TNF was dispersed in anhydrous CB (0.5 mg/mL) through ultrasonication for 45 minutes, using an Elmasonic S30H sonication bath. Afterwards, the dispersion was centrifuged at 4200 rpm for 30 minutes and a concentrated supernatant (GO-TNF ink) was isolated and decanted. The final GO-TNF ink was used without further purification. The solvent's selection (anhydrous CB) for GO-TNF ink preparation was done by taking into account that the binary blend (PTB7:PC$_{71}$BM) of the active layer of the inverted OSC was also dissolved in anhydrous CB.

3. Device fabrication process

The inverted OSC devices were fabricated onto 20 mm × 15 mm indium-tin-oxide (ITO) glass substrates, exhibiting sheet resistance (R_s) value of 20 Ω sq^{-1}. Next, a ~10 nm thick PFN (0.5 mg/ml, in 1 ml of MeOH and 2 μl of AcOH) layer (ETL) was spin-cast at 1000 rpm for 45 sec followed by thermal annealing at 150 °C for 30 sec. Then the photoactive layer consisting of PTB7:PC$_{71}$BM at a 1:1.5 (10 mg:15 mg) ratio, dissolved in CB and 3% 1,8-diiodooctane (DIO) was spin-coated at 1500 rpm on top of the PFN layer. In case of GO-TNF ternary blends, GO-TNF ink was directly added in several volume ratios into the binary PTB7:PC$_{71}$BM blend and mixed for 6h to form a homogeneous solution. Finally, MoO_3 HTL layer (8 nm) and the top Al electrode (100 nm) were thermally evaporated through a shadow mask, defining an active area of 4 mm^2 for each device.

4. GO-TNF thin films preparation for microscopic characterization

4.1 Cleaning process of glass substrates

The glass substrates were cleaned using a 5-step cleaning process. Firstly, the glass substrates were sonicated into an aqueous solution of detergent for 15 minutes. Afterwards, the residual amount of aqueous detergent solution was wiped and blown away from the glass substrates. Subsequently, the same procedure was followed using different solvents with the following order: 1) deionized water 2) acetone 3) isopropanol. Then the glass substrates were dried in the oven for 30 minutes. Thereafter, the glass substrates underwent a UV-ozone treatment in an M-BRAUN glovebox in order to be more furtherly purified and for their hydrophilicity to be increased.

4.2 GO-TNF thin films fabrication

Immediately after the UV-ozone treatment of the 5-step cleaning process, the glass substrates were sprayed with an infinity CR Plus spray gun filled with 4 ml of the wanted dispersion. The spray gun was mounted onto a custom made, automatically moving axis, 12 cm above the glass substrates which were constantly heated at 180 °C. After each spray cycle, the substrates were given one minute in order for the solvent to be evaporated. The pressure of the air that was fed into the spray gun was 2 bar.

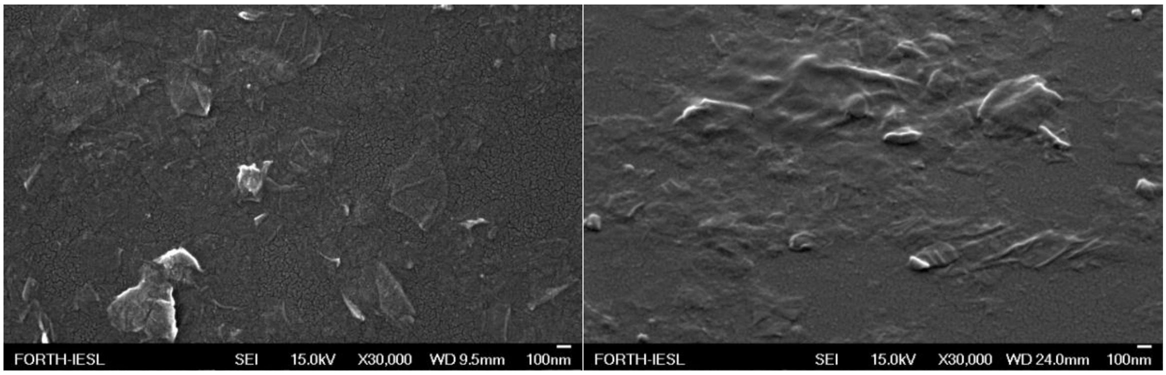

Figure S11. FE-SEM images of GO-TNF.

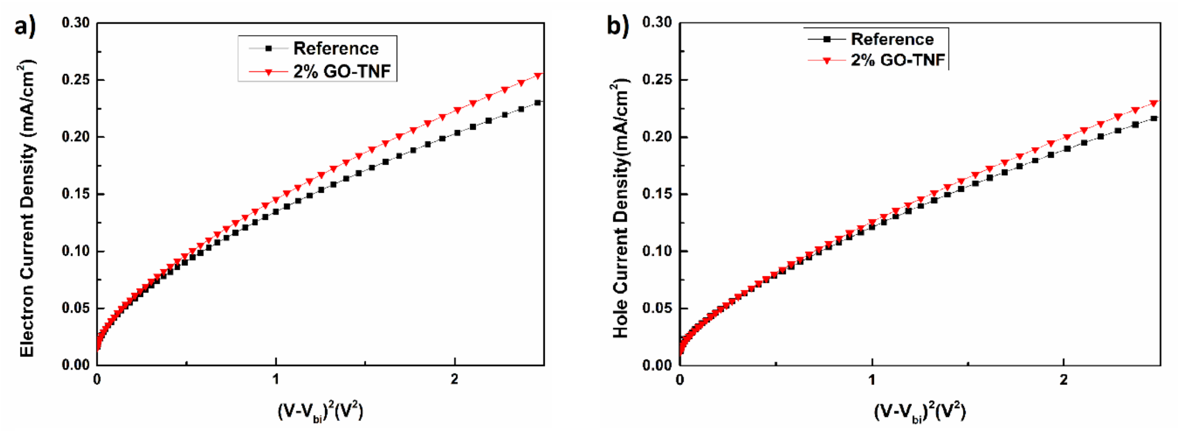

Figure S12. *J-V*^{2} characteristics of the fabricated **a)** electron-only and **b)** hole-only devices. The black line refers to the control device (PTB7:$PC_{71}BM$), while the red line corresponds to the ternary PTB7:GO-TNF (2%):$PC_{71}BM$.

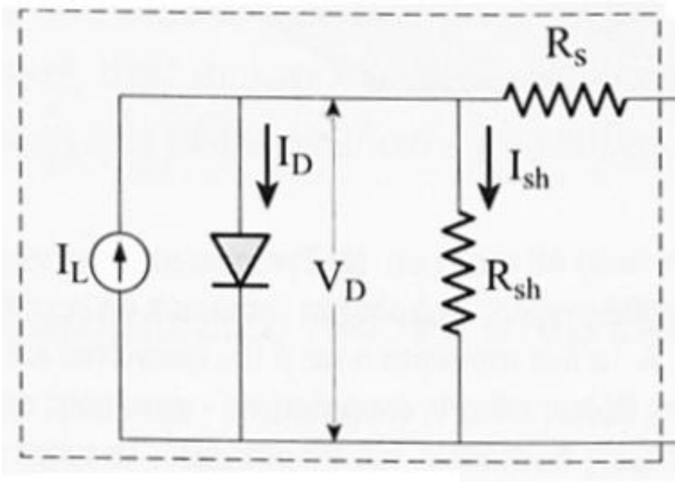

Figure S13. The one diode equivalent circuit model corresponding to the ternary OSC device incorporating 2% GO-TNF ink.

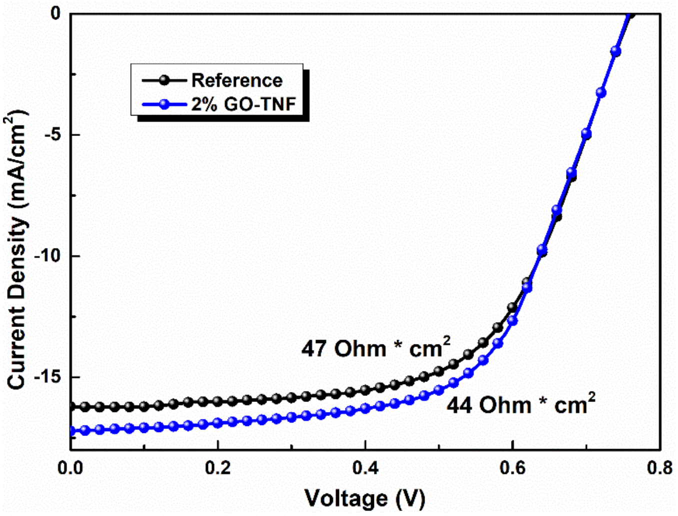

Figure S14. The effect of shunt resistance.
